# Competing Photoisomerization and Excited-State Intramolecular Proton Transfer in a Visible-Light-Driven Salicylidene Schiff Base Functionalized Molecular Motor: Insights from Ultrafast Nonadiabatic Dynamics Simulations

**DOI:** 10.3390/ijms27156627

**Published:** 2026-07-25

**Authors:** Xiaojuan Pang, Yue Yan, Xueyan Cui, Ningbo Zhang

**Affiliations:** 1 School of Materials Science and Physics, China University of Mining and Technology, Xuzhou 221116, China; ts25180014a31@cumt.edu.cn; 2 School of Mines, China University of Mining and Technology, Xuzhou 221116, China; 3 Department of Science, Henan Institute of Technology, Xinxiang 453003, China; cxy@hait.edu.cn

**Keywords:** visible-light-driven molecular motor, photoisomerization, excited-state intramolecular proton transfer (ESIPT), nonadiabatic molecular dynamics, ultrafast photodynamics

## Abstract

We investigate the ultrafast dynamics of a visible-light-driven salicylaldehyde Schiff base-functionalized molecular motor using non-adiabatic molecular dynamics (NAMD) at the orthogonalization-corrected method 2 and the multireference configuration interaction (OM2/MRCI) level. Results reveal a dynamic competitive interplay between E → Z photoisomerization and excited-state intramolecular proton transfer (ESIPT). Importantly, we reveal a direct coupling mechanism between double-bond rotation and proton transfer: within a critical 500–900 fs window, ESIPT transiently stalls rotor rotation while modulating the central double-bond order. The S_1_ state lifetime is ~1677 fs. Time-resolved fluorescence analysis further predicts rapid fluorescence quenching within ~37 fs accompanied by a pronounced spectral red-shift, indicating that the system quickly enters a dark state with negligible oscillator strength. This study provides atomistic insights into the relaxation pathways of visible-light-driven motors, offering a theoretical basis for designing efficient long-wavelength responsive molecular machines.

## 1. Introduction

Light-driven molecular motors have attracted significant interest due to their ability to perform directional mechanical motion at the molecular scale. As key components of artificial molecular machines, they convert light energy into controlled unidirectional rotation, enabling dynamic and responsive behavior in molecular systems [1,2]. The 2016 Nobel Prize in Chemistry was awarded to the field of artificial molecular machines, marking the official entry of molecular machine research into a phase of rapid development [1]. Compared to traditional static molecular systems, molecular motors can not only undergo conformational changes but also achieve energy transfer, chiral regulation, and dynamic functional output through continuous rotation [3]. Consequently, they exhibit significant application prospects in fields such as smart materials [4,5], supramolecular chemistry [6], and nanomedicine [7,8].

Currently, light-driven molecular motors based on overcrowded alkene structures represent the most extensively studied class of systems. Owing to their excellent dynamic chirality features, these systems offer unique advantages in areas such as chirality transfer, liquid crystal tuning, dynamic supramolecular assembly, and stimuli-responsive materials [9]. However, most conventional first-generation molecular motors rely on ultraviolet (UV) light driving, which somewhat limits their further applications in biological systems and functional materials [2,10]. UV light not only suffers from limited penetration depth but can also induce molecular degradation and biological damage. Consequently, developing visible-light-responsive molecular motors has emerged as a crucial research direction in this field.

In order to achieve a red-shift in the absorption spectra of molecular motors, researchers have developed various molecular design strategies, such as expanding the π-conjugated system, building push–pull electronic structures [11], introducing metal coordination, and utilizing energy transfer sensitization [12]. Because of their strong intramolecular hydrogen bonding, adjustable OH/NH tautomeric equilibrium, and exceptional π-electron delocalization ability, salicylidene Schiff bases have been found to have extensive applications in visible-light-responsive molecular switches and photofunctional materials [13]. Based on these features, a recent work published in Nature Communications introduced the salicylidene Schiff base structural unit into the backbone of a first-generation molecular motor, formally named (Z)-1,1′-bis(2,7-dimethyl-6-hydroxy-5-((phenylimino)methyl)-2,3-dihydro-1H-inden-1-ylidene) (hereafter referred to as M1), successfully creating a series of completely visible-light-driven molecular motors. This strategy enabled a remarkable red-shift of about 100 nm in the absorption peak compared to traditional systems, while maintaining good unidirectional rotation and a high photostationary state (PSS). Additionally, the system shows clear solvent and temperature dependence, and its photoisomerization is heavily affected by the OH/NH tautomerization process. This indicates that there might be a competitive relationship between ESIPT and double-bond rotation within the molecule [2].

Despite experimental evidence confirming the efficient visible-light-driven rotation of these molecular motors, their excited-state ultrafast dynamic mechanisms remain poorly understood. Several key questions still lack clear theoretical explanations: how electronic excitation drives molecular backbone twisting post-excitation, how conical intersections participate in non-radiative decay, whether there is a dynamic competition between excited-state proton transfer and molecular rotation [14], and how electronic state coupling influences the photoisomerization quantum yield. Consequently, employing NAMD simulations to explore their excited-state evolution at the atomic level is crucial for unraveling the photochemical mechanisms underlying these visible-light-driven molecular motors [15].

NAMD methods can simultaneously describe the coupling between electronic and nuclear motions, making them particularly suitable for investigating complex excited-state decay processes near conical intersections. Among these, dynamics simulations based on the Fewest-Switches Surface Hopping (FSSH) [16] theoretical framework have been widely applied to study photoisomerization, excited-state proton transfer, and the rotational mechanisms of molecular motors. Through trajectory analysis, researchers can not only obtain excited-state lifetimes, electronic state population evolution, and nonadiabatic transition behaviors, but also further reveal the structural changes and energy dissipation pathways of molecules on ultrafast timescales. Therefore, performing nonadiabatic dynamics studies on this class of salicylaldehyde Schiff base-functionalized molecular motors will facilitate a profound understanding of the excited-state decay mechanisms of visible-light-driven molecular motors, thereby providing a solid theoretical foundation for the design of novel high-efficiency, long-wavelength responsive molecular machines.

## 2. Results and Discussion

### 2.1. Ground-State Configurational Characteristics and Structural Evolution During Photoinduced Z/E Isomerization

**Figure 1:**
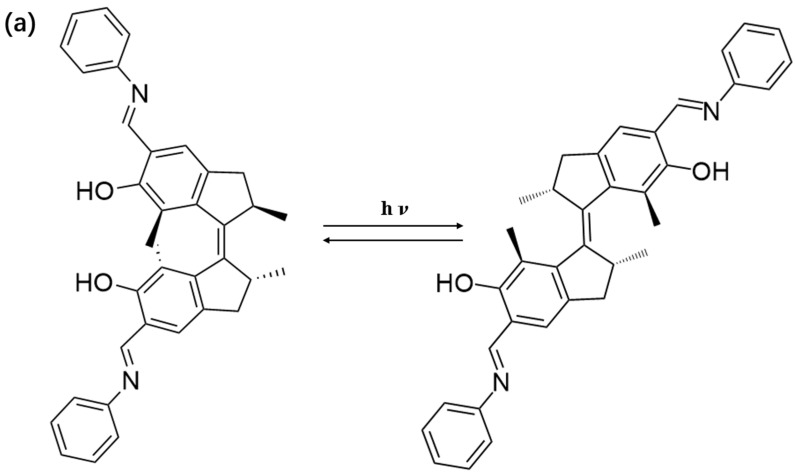

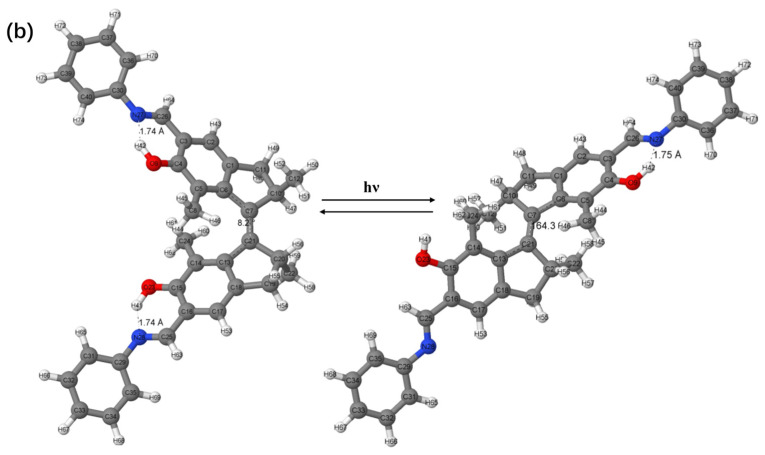
(**a**) Schematic representation of the photoinduced E–Z isomerization of the molecular motor. (**b**) Optimized ball-and-stick structures of the E and Z isomers with selected intramolecular hydrogen-bond distances (Å) and dihedral angles (°).

In this study, the high-precision semiempirical multireference OM2/MRCI method was employed to perform ground-state (S_0_) geometric optimization and photoisomerization analysis of the salicylaldehyde Schiff base molecular motor system. The molecular moiety below the central double bond (C7 = C21) is defined as the relatively stationary “stator,” whereas the upper moiety constitutes the rotatable “rotor.” As depicted on the left side of Figure 1a, the optimized ground-state equilibrium geometry exhibits a Z-configuration. As shown in Figure 1b, its central dihedral angle (C6–C7–C21–C13) is 8.2°, indicating that this isomer possesses a highly quasi-planar geometric feature in the ground state. Within this coplanar conformation, a strong intramolecular hydrogen bond (O-H···N) is formed between the lone pair of the imine nitrogen atom (N) and the phenolic hydroxyl hydrogen atom (H) inside the respective salicylaldehyde Schiff base fragments of both the stator and rotor. The hydrogen bond length is approximately 1.74 Å, which further thermodynamically stabilizes the planarity of this configuration. Upon photoinduction (hν) at a specific wavelength, the molecule overcomes the excited-state potential barrier, undergoes nonadiabatic dynamic evolution, and relaxes back to the ground state, during which the central double bond twists to yield the E-configured isomerization product shown on the right side of Figure 1a. Notably, the conformational inversion leads to a drastic expansion of the central dihedral angle from the initial 8.2° to 164.3°. This Z-to-E geometric distortion triggers significant steric hindrance, forcing the imine group of the rotor moiety to deviate severely from its initial position. Accompanied by this dramatic spatial conformational displacement, the distance between the hydrogen donor and acceptor substantially exceeds the interaction threshold, ultimately leading to the complete disruption of the original intramolecular hydrogen-bonding network. The optimized geometries of the relevant structures are provided in Section S1 of the Supporting Information.

**Figure 2:**
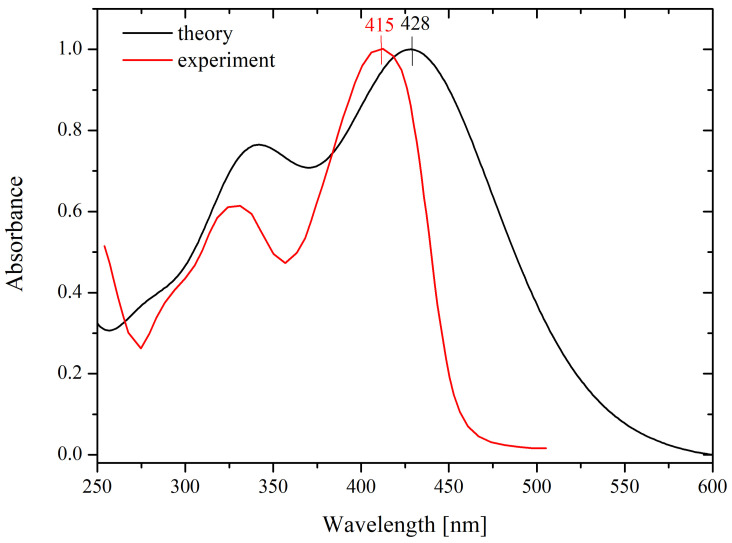
Simulated and experimental UV–Vis absorption spectra of the molecular motor. The theoretical spectrum (black solid line) was calculated at the TDDFT level, and the experimental spectrum (red solid line) [2] is shown for comparison.

To assess the reliability of the present computational methodology, we calculated the electronic absorption spectrum using TDDFT and compared it with the experimental UV–Vis spectrum reported by van Vliet et al. [2]. While the nonadiabatic dynamics simulations were performed at the OM2/MRCI level, the absorption spectrum was computed using TDDFT because this method is computationally efficient for spectral simulations and has been shown to provide vertical excitation energies with accuracy comparable to that of OM2/MRCI for organic chromophores. As shown in Figure 2, the TDDFT-calculated spectrum reproduces the overall lineshape of the experimental absorption band reasonably well. The theoretical maximum absorption wavelength (λ_max_) is predicted at 428 nm, in good agreement with the experimental value of approximately 415 nm, with a deviation of only 13 nm. This small discrepancy falls within the typical error range of TDDFT calculations for such push–pull systems and confirms that the adopted computational protocol adequately captures the electronic excitation characteristics of the molecular motor. We note that the experimental study did not report excited-state lifetimes or photoisomerization quantum yields, which precludes direct quantitative validation of these dynamical observables. Nevertheless, the good agreement between the calculated and measured absorption spectra lends confidence to the theoretical description of the electronic structure and provides a solid basis for the subsequent nonadiabatic dynamics simulations. Future ultrafast transient absorption spectroscopy, time-resolved fluorescence measurements, and photoisomerization quantum-yield determinations would provide valuable benchmarks for further validating the excited-state dynamics predicted in this work.

### 2.2. Time-Dependent Average Populations of S_0_ and S_1_

**Figure 3:**
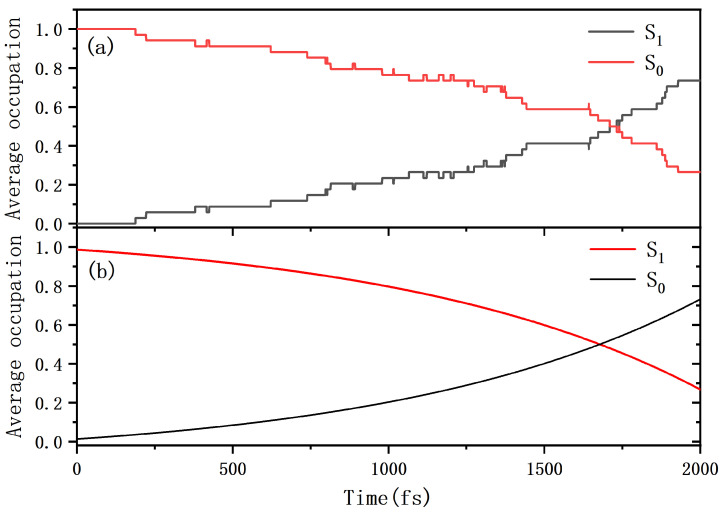
Time evolution of the average populations of S_0_ and S_1_. (**a**) Results from nonadiabatic dynamics simulations. (**b**) Single-exponential fitting of the population dynamics.

To thoroughly investigate the dynamical behavior of the salicylaldehyde Schiff base molecular motor M1, excited-state nonadiabatic molecular dynamics simulations were performed. Figure 3a presents the time evolution of the average populations of the ground state (S_0_, black) and the first excited state (S_1_, red) obtained from all trajectories. Owing to the finite number of trajectories, the averaged populations exhibit a characteristic stepwise behavior, reflecting individual surface-hopping events accumulated over time. Although both E/Z photoisomerization and excited-state intramolecular proton transfer (ESIPT) occur during the excited-state evolution, these processes mainly correspond to different nuclear relaxation pathways on the S_1_ potential energy surface rather than independent electronic-state decay channels. Consequently, despite possible deviations from ideal single-exponential behavior, the ensemble-averaged S_1_ population exhibits an overall smooth decay within the present trajectory statistics. Therefore, a single-exponential function was adopted as a phenomenological description of the population evolution. The population evolution shown in Figure 3a was fitted using a single-exponential function, y = y_0_ + A_1_ × exp(x/t_1_), and the resulting smooth fitting curves are presented in Figure 3b. The fitting yielding A_1_ = 0.07349. t_1_ = 1677 fs. Upon photoexcitation at t = 0 fs, the system initially populates the FC region of the S_1_ state. Following structural relaxation on the S_1_ surface, the S_1_ state population monotonically declines while the S_0_ population rises. The two curves intersect at approximately 1677 fs, at which point the average populations of the two states become equal (0.5)—i.e., roughly half of the ensemble has undergone nonadiabatic transitions from the excited state to the ground state. This triggers surface hopping events that return the system from the excited state to the ground state, establishing an excited-state lifetime of about 1677 fs.

Although both ESIPT and photoisomerization occur on the S_1_ potential energy surface, these processes represent distinct nuclear relaxation pathways within the same electronic state rather than separate electronic-state decay channels. Therefore, the ensemble-averaged S_1_ population decay can be effectively described by a phenomenological single-exponential function, and the extracted lifetime should be interpreted as an effective parameter characterizing the overall depopulation timescale of the S_1_ state, rather than a rigorous kinetic constant associated with a single elementary process. This simplified treatment provides a convenient measure of the excited-state lifetime while acknowledging the underlying mechanistic complexity.

### 2.3. Dynamic Competition Between Photoisomerization and Proton Transfer

**Figure 4:**
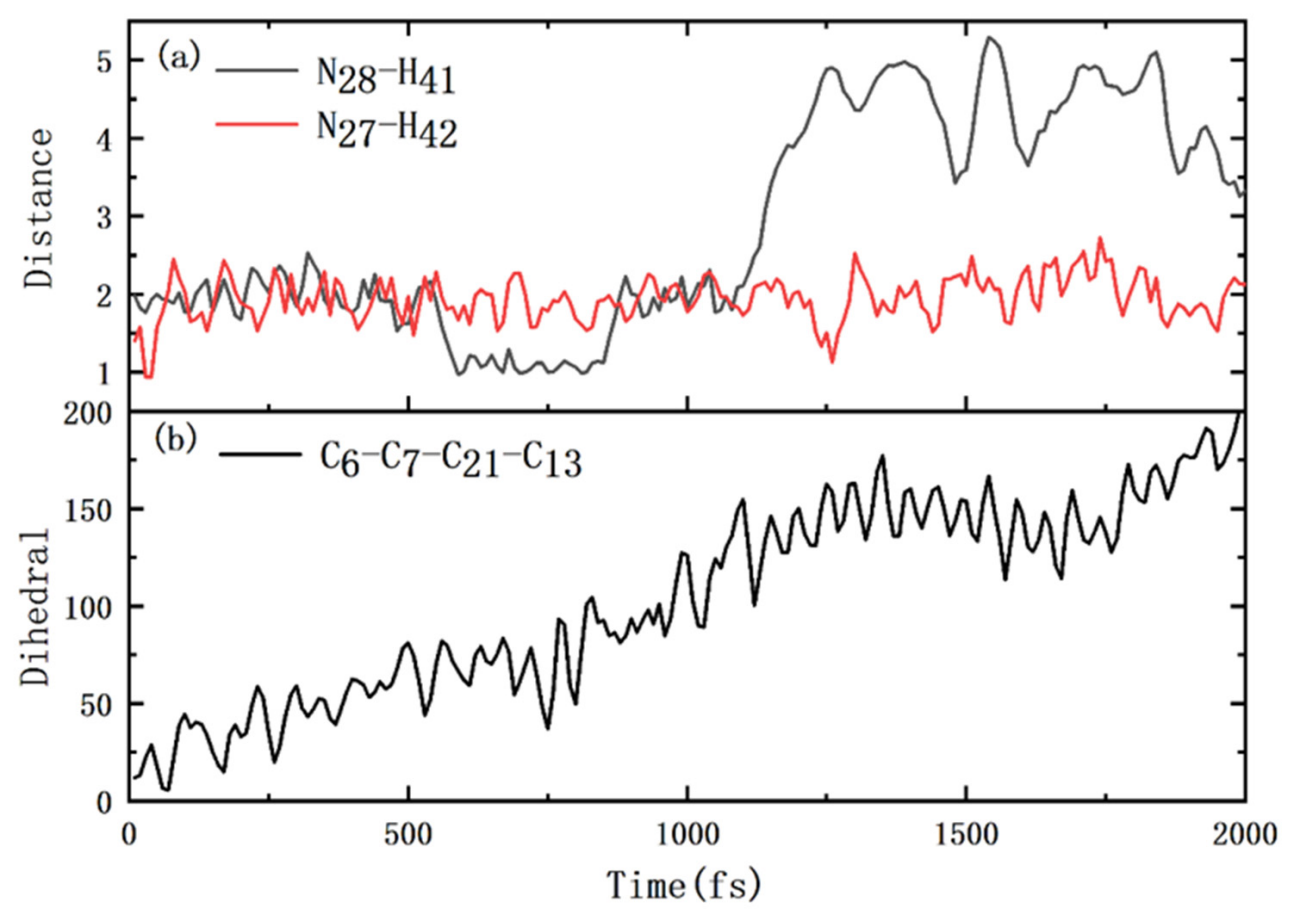
(**a**) Time evolution of the distances between N28–H41 and N27–H42. (**b**) Evolution of the dihedral angle C6–C7–C21–C13 as a function of time.

To elucidate the detailed nonadiabatic evolution mechanism of the M1 molecular motor following photoexcitation, we tracked the temporal evolution of geometric parameters along a typical successful trajectory, revealing the dynamic coupling between photoisomerization-related torsional motion and ESIPT. The time evolution of the central dihedral angle (C6–C7–C21–C13) visually demonstrates the rotation of the rotor relative to the stator, as depicted in Figure 4b. Initially, the motor resides in a nearly planar, ground-state stable configuration with a dihedral angle of approximately 8.2°. Upon photoexcitation, the dihedral angle increases in an oscillatory manner over time, indicating that the motor overcomes steric hindrance and begins to rotate. It reaches an equilibrium state around 1500 fs and subsequently oscillates around this equilibrium angle until the end of the simulation. Furthermore, we extracted the temporal variation in the N–H bond length. As shown in Figure 4a, during the 500–900 fs interval, the N28-H41 distance exhibits a pronounced decrease, reaching a minimum of approximately 1.1 Å. This indicates the occurrence of proton transfer, where H41 migrates from the O atom to the N28 atom. The resulting excited-state keto tautomer is generally lower in energy and more stable than the initial excited-state enol form. Concurrently, we observed that during this 500–900 fs window—the exact period of proton transfer—the C6–C7–C2–C13 dihedral angle oscillates back and forth around 65°. The transient stalling observed during the 500–900 fs interval indicates that the torsional motion does not proceed continuously while ESIPT takes place. Instead, the rotor temporarily oscillates around a dihedral angle of approximately 65°, suggesting that the proton-transfer motion transiently perturbs the rotational progression. This behavior is consistent with a dynamical competition between ESIPT and double-bond rotation during the excited-state relaxation process, which may reduce the probability of successful E → Z photoisomerization. Crucially, this transient modulation of the torsional coordinate suggests that proton transfer influences the early-stage photoisomerization evolution and may contribute to the reduced photoisomerization efficiency observed from the trajectory ensemble. These results provide compelling atomistic evidence for the experimental observation of new spectral bands in polar protic solvents [13].

It is worth noting that, in the same representative trajectory, we also observed a transient proton-transfer event involving the N27–H42 distance during the 1000–1250 fs window. As shown in Figure 4a, the N27–H42 distance decreases briefly within this time period, indicating a temporary proton migration. However, this proton transfer is short-lived; as the structural evolution proceeds, the strong polarity of the oxygen atom drives a rapid reverse proton transfer (back proton transfer), restoring the original hydrogen-bonding configuration within a few hundred femtoseconds. Consequently, the N27–H42 distance returns to its initial value, and this transient event does not lead to a stable keto tautomer or contribute to productive photoisomerization. This observation further supports our conclusion that ESIPT and double-bond rotation are dynamically competitive rather than cooperative: although proton transfer can occur transiently during the rotational process, it is often reversed when it does not align with the favorable conformational evolution toward the conical intersection.

In addition to the representative trajectory discussed above, we observed similar reverse proton transfer events in several other trajectories, indicating that proton transfer in this system is dynamically reversible rather than unidirectional. In these trajectories, ESIPT occurs transiently but is subsequently reversed via back proton transfer, restoring the original enol configuration. This observation further supports the competitive nature of the ESIPT–isomerization interplay and highlights the complexity of the excited-state potential energy surface, where multiple proton-transfer pathways and reverse processes coexist. The reversible nature of ESIPT suggests that proton transfer does not necessarily commit the system to a specific relaxation pathway; instead, the ultimate fate of the excited-state population depends on the interplay between the proton-transfer coordinate and the rotational coordinate, as well as the accessibility of conical intersection regions. These findings underscore the importance of trajectory-level analysis in uncovering the mechanistic diversity of photochemical reactions in complex molecular systems.

**Figure 5:**
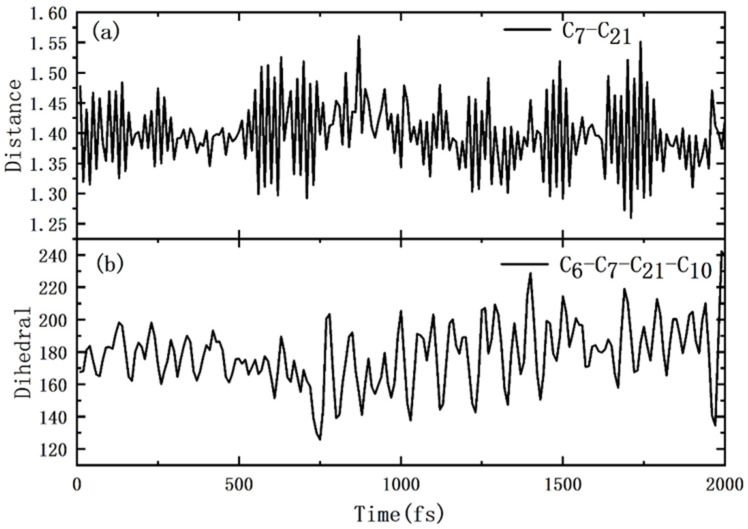
(**a**) Time evolution of the C7–C21 bond distance. (**b**) Evolution of the dihedral angle C6–C7–C21–C10 as a function of time.

In addition to the central dihedral angle (C6–C7–C21–C13), we also extracted the pyramidalization dihedral angle (C6–C7–C21–C10) to characterize the concerted motion of the backbone surrounding the rotor. As shown in Figure 5b, this pyramidalization angle predominantly oscillates within the range of 160–200°, indicating that the local framework generally maintains a planar configuration. A relatively distinct deviation is observed around 700 fs. However, this change is transient, and the system rapidly reverts to its original oscillatory regime. This demonstrates that while the rotor undergoes rotation, the adjacent backbone does not experience a synchronous, large-scale rearrangement. Instead, it responds to the global structural evolution through a restricted twisting motion.

Concurrently, we analyzed the time evolution of the central C7–C21 bond length, as depicted in Figure 5a. It can be seen that the central C=C double bond length is predominantly distributed between 1.35 and 1.45 Å, oscillating around an average of ~1.4 Å. During the 600–900 fs interval, a noticeable stretching of the bond is observed, though it rapidly retracts to its average value. Combining this with the previously discussed evolution of the central dihedral angle, it becomes evident that within the 500–900 fs time window, the system simultaneously experiences proton transfer and hindered rotor rotation. During this specific stage, the C6–C7–C21–C10 dihedral angle oscillates mainly within the range of 130–180°, accompanied by intensified fluctuations in the C7–C21 bond length. This distinct phenomenon highlights a significantly enhanced local structural response of the system during the proton transfer window.

**Figure 6:**
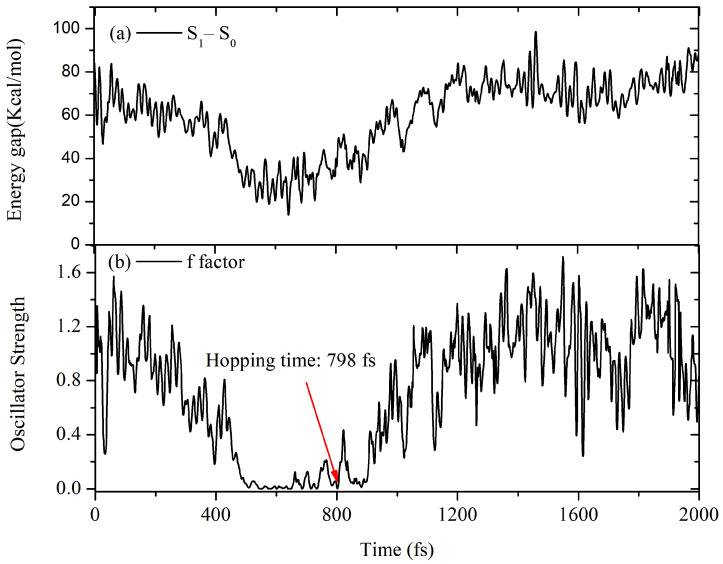
(**a**) Time-dependent energy gap of S_1_–S_0_ state and (**b**) time evolution of oscillator strength along a representative trajectory.

To further characterize the electronic-structure evolution underlying the observed dynamical competition, we extracted the time-dependent S_1_–S_0_ energy gap and the S_1_ → S_0_ oscillator strength along a representative trajectory, as shown in Figure 6. As depicted in Figure 6a, immediately following photoexcitation, the energy gap between the S_1_ and S_0_ states exhibits pronounced fluctuations with an average value of approximately 35–46 Kcal/mol (1.5–2.0 eV). As the trajectory evolves, the energy gap gradually decreases, approaching the S_1_/S_0_ conical intersection seam where nonadiabatic transitions become feasible. Notably, during the 500–900 fs window—coinciding with the ESIPT event and transient stalling of the rotor rotation identified in Figure 4—the energy gap shows a discernible narrowing, suggesting that proton transfer modulates the energetic proximity of the two electronic states and may facilitate subsequent surface-hopping events.

**Figure 7:**
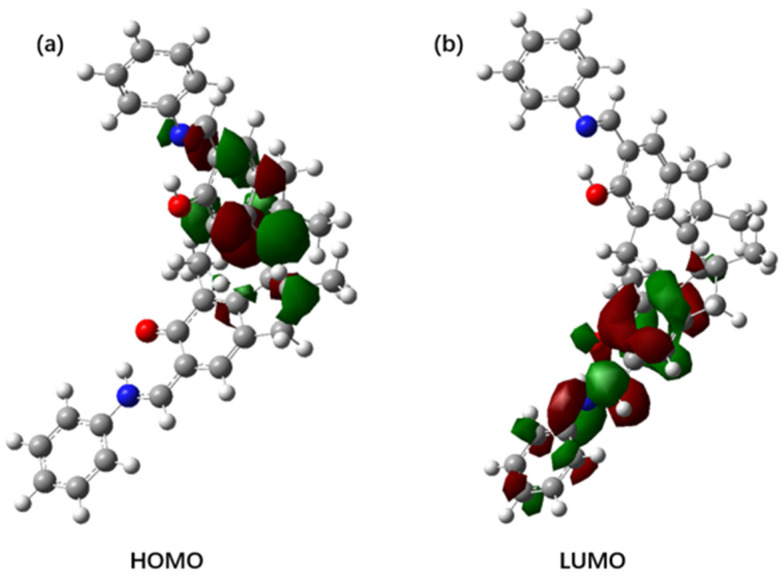
Visualization of the frontier molecular orbitals (FMOs) of the molecule, including (**a**) the highest occupied molecular orbital (HOMO) and (**b**) the lowest unoccupied molecular orbital (LUMO). The differently colored isosurfaces indicate opposite phases of the molecular orbital wavefunction.

**Figure 8:**
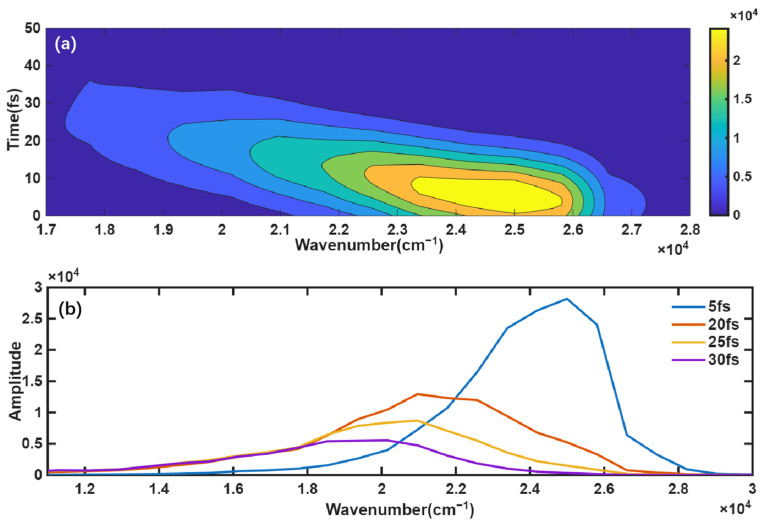
Time-dependent fluorescence emission spectra of excited state dynamics. (**a**) Time-resolved deconvoluted emission spectrum. (**b**) The wavelength-resolved decay information at selected time delays, showing the evolution of spectral shape and intensity.

Figure 6b displays the evolution of the S_1_ → S_0_ oscillator strength along the same representative trajectory. Following vertical excitation to the FC region at t = 0 fs, the oscillator strength retains a relatively high value, consistent with the bright nature of the initial photoexcitation. However, upon structural relaxation away from the FC region, the oscillator strength rapidly decreases within a few hundred femtoseconds, eventually approaching near-zero values for most of the remaining excited-state lifetime. This dramatic reduction in oscillator strength indicates that the molecule quickly evolves into a configuration region with negligible radiative transition probability—effectively a “dark” region of the S_1_ potential energy surface. The molecule initially possesses a relatively high oscillator strength at the FC region (t = 0 fs), consistent with the frontier molecular orbital analysis shown in Figure 7. Subsequently, the rapid quenching of oscillator strength is fully consistent with the time-resolved fluorescence spectra presented in Figure 8, where the fluorescence signal decays within approximately 37 fs. Importantly, the oscillator strength remains persistently low even when the trajectory undergoes subsequent structural rearrangements, demonstrating that once the system departs from the FC region, the radiative channel remains effectively shut off for the remainder of the excited-state evolution. This observation corroborates our previous conclusion that, apart from the initial ultrafast fluorescence component near the FC region, the S_1_ excited-state decay is predominantly governed by nonradiative relaxation pathways. Consequently, the 1677 fs excited-state lifetime primarily reflects the time required for the system to evolve toward the S_1_/S_0_ conical intersection, rather than the duration of fluorescence emission. Together, the energy-gap narrowing and oscillator-strength quenching provide compelling electronic-structure evidence for the dynamical competition mechanism: as the rotor twists and proton transfer proceeds, the system is funneled toward a region of strong nonadiabatic coupling with simultaneously suppressed radiative character, ultimately dictating the photoisomerization quantum yield and the excited-state deactivation pathway. The NVE ensemble conservation and the NACV-rescaling artifacts, including transient jumps in angular momentum and temperature (Section S2, Figure S1), are discussed in the Supporting Information.

### 2.4. Frontier Molecular Orbital Analysis and Excited-State Dynamics

To further understand the aforementioned dynamical behaviors from the perspective of electronic structure, the frontier molecular orbital (FMO) distributions (HOMO and LUMO) of the molecule at its ground-state equilibrium configuration were analyzed, as illustrated in Figure 7. The HOMO is continuously distributed across multiple atoms over the C=N bond and its adjacent aromatic conjugated region, indicating that the π-electrons are highly delocalized across the molecular framework. In contrast, the LUMO electron density is distinctly concentrated on the lower half of the molecule—specifically on the stator region and its neighboring conjugated backbone—exhibiting a pronounced spatial separation feature. This orbital distribution pattern is in excellent agreement with the observed dynamics. On one hand, photoexcitation weakens the π-bonding character of the central double bond, thereby lowering the rotational barrier of the rotor, which perfectly matches the gradual increase in the central dihedral angle. On the other hand, the substantial distribution of the HOMO in the O-H···N region, along with its spatial extension between the donor and acceptor, reveals a strong orbital interaction that stabilizes the intramolecular hydrogen bond. The photoinduced electronic redistribution alters the local electronic environment of the O-H···N network, promoting proton transfer to the nitrogen atom and thus explaining the ESIPT phenomenon observed within the 500–900 fs time window. Ultimately, this significant shift in electron density from the rotor to the stator moiety highlights a prominent intramolecular charge transfer (ICT) characteristic.

Such redistribution of electron density attenuates the degree of electron delocalization within the central conjugated system, thereby altering the local electronic structure near the central double bond and further dictating the subsequent conformational evolution. In conjunction with the preceding dynamical results, it can be deduced that the excited-state electron transfer not only impacts the stability of the central double bond but also exerts a synergistic influence on both proton transfer and rotor motion by modulating the local hydrogen-bonding environment. Statistically, among the 34 fully completed trajectories, 26 underwent proton transfer (accounting for ~76.4%), and 10 successfully underwent E-Z isomerization (~29.4%). Notably, 7 out of the 34 trajectories (~20.6%) accomplished both proton transfer and successful isomerization processes.

To establish a clearer correlation between ESIPT and rotational success, we further analyzed the isomerization yields within different trajectory subsets. As summarized in Table 1, among the 26 trajectories that underwent ESIPT, 7 successfully completed the E → Z isomerization, yielding a success rate of ~26.9%. In contrast, among the remaining 8 trajectories where ESIPT did not occur, 3 achieved successful isomerization, representing a higher success rate of ~37.5%. A more detailed trajectory-by-trajectory correlation analysis, including the timing of ESIPT events and isomerization completion for all 29 fully propagated trajectories, is provided in Section S3 of the Supporting Information (Figure S2 and Table S1). Although the relatively small sample size, inherent to computationally demanding multireference nonadiabatic dynamics simulations, limits rigorous statistical significance analysis, the observed reduction in isomerization yield in the presence of ESIPT suggests that ESIPT may influence the excited-state relaxation dynamics. Specifically, the lower isomerization yield associated with ESIPT is consistent with the possibility that proton transfer competes with double-bond rotation during excited-state deactivation. However, the present trajectory statistics alone are insufficient to establish this competitive mechanism conclusively, and further validation using more accurate electronic structure methods and more extensive nonadiabatic dynamics simulations will be required.

### 2.5. Time-Resolved Fluorescence Spectral Evolution Following Photoexcitation

To further unveil the theoretical deactivation mechanism of the molecular motor, time-resolved fluorescence emission spectra were simulated, mapping wavenumber (x-axis) against time (y-axis) with fluorescence intensity indicated by a color scale. Figure 8a clearly visualizes an ultrafast decay of the fluorescence signal. By ~37 fs, the high-intensity region entirely dissipates, indicating a rapid decrease in ensemble-averaged fluorescence intensity. This early fluorescence attenuation reflects the rapid structural dispersion of the excited-state ensemble away from the FC region, whereas the oscillator strength evolution along individual trajectories in Figure 6b shows that some configurations continue to undergo further radiative suppression on a longer femtosecond timescale. This observation strongly corroborates our trajectory simulations. While the overall S_1_ lifetime was determined to be ~1677 fs, the emission spectrum demonstrates a radiative cessation at just 37 fs. The simulated fluorescence spectra indicate that the fluorescence intensity decays rapidly within approximately 37 fs, suggesting that the ensemble of trajectories quickly evolves away from the Franck–Condon region into configurations with substantially reduced radiative transition probability. Essentially, as the molecule structurally relaxes away from the FC region, the S_1_ → S_0_ oscillator strength plummets to near zero, rendering the system radiatively silent for most of its excited-state lifespan. Following the initial photoexcitation to the S_1_ FC region, the system undergoes rapid structural relaxation on the excited-state surface. To capture these early-stage relaxation dynamics, Fourier transformation was employed to derive the frequency-domain spectra at various times. Figure 8b extracts emission cross-sections at key intervals from 5 fs to 30 fs, using log-normal function fitting to map curve amplitudes to relative oscillator strengths. As time progresses, the emission peaks suffer a dramatic amplitude reduction alongside a striking spectral red-shift. Specifically, the central wavenumber shifts from roughly 2.5 × 10^4^ cm^−1^ at 5 fs to 1.9 × 10^4^ cm^−1^ at 30 fs. This synchronous red-shifting and intensity attenuation perfectly visualizes the rapid descent of the excited-state wavepacket down the S_1_ potential energy surface, where the concurrent geometric distortions severely diminish the ground-to-excited-state transition dipole moment.

## 3. Materials and Methods

In this work, the photoisomerization dynamics of a hydrazone-based molecular switch were investigated, employing NAMD combined with the semiempirical OM2/MRCI approach. All electronic structure and dynamics calculations were performed utilizing the MNDO99 program package (version 7.0). The OM2 method is highly capable of describing the electronic structure characteristics of organic conjugated systems, while the MRCI approach effectively addresses the significant electron correlation effects inherent in excited-state processes. Consequently, this combined methodology is highly suitable for simulating complex photochemical reactions [17]. 

The excited-state dynamics were simulated using the FSSH method proposed by Tully [16]. By introducing a stochastic hopping mechanism between different electronic potential energy surfaces, this approach captures the coupled evolution of electronic and nuclear motions, thereby effectively describing the nonadiabatic transition behaviors near conical intersections. During the dynamics propagation, the electronic state hopping probabilities are computed based on the nonadiabatic coupling strengths, and stochastic sampling is subsequently employed to determine whether an electronic state switch occurs. In the electronic structure calculations, potential energies, energy gradients, and nonadiabatic coupling vectors were all evaluated analytically to enhance the stability and accuracy of the dynamics simulations. When a successful surface hop occurs, velocity rescaling is performed along the nonadiabatic coupling vector (NACV) direction to conserve total energy. For downward hops, the excess potential energy is converted into kinetic energy by rescaling the velocity component along the NACV. The self-consistent field (SCF) calculations were performed within the restricted open-shell Hartree–Fock (ROHF) framework [18]. For the MRCI calculations, the active space comprised 16 electrons distributed over 16 orbitals, consisting of the nine highest occupied molecular orbitals (HOMOs) and the seven lowest unoccupied molecular orbitals (LUMOs), including two singly occupied orbitals, thereby ensuring an adequate description of the key excited-state configurations. The active space was constructed by including orbitals sequentially according to their orbital energy ordering, starting from the HOMO and proceeding downward for occupied orbitals and upward for unoccupied orbitals, without manual intervention. Within this active space, the excitation level was restricted to single excitations (CIS), providing an accurate description of the excited-state dynamics while maintaining computational feasibility. Notably, the empirical decoherence correction proposed by Truhlar et al. [19] and Granucci et al. [20] to enhance the fewest switches scheme’s internal consistency was not employed in this study. This may result in an overestimation of electronic coherence and influence the quantitative population dynamics and excited-state lifetimes, although the qualitative mechanistic picture is generally expected to remain unchanged.

The ground-state structures of the E- and Z-isomers were first optimized using density functional theory (DFT) with the B3LYP functional [21,22]. Based on the optimized ground-state geometries, the electronic absorption spectrum was calculated using time-dependent density functional theory (TDDFT) at the same level of theory and compared with the available experimental spectrum to validate the computational methodology. The resulting geometries were further re-optimized at the OM2/MRCI level [23,24]. Subsequently, initial nuclear coordinates and momenta were sampled based on the Wigner distribution at 300k [25,26], to construct initial conditions that satisfy quantum vibrational statistical characteristics, yielding a series of initial configurations with distinct coordinates and velocities. The subsequent nonadiabatic molecular dynamics simulations were performed under the microcanonical (NVE) ensemble without applying a thermostat, and the total energy was monitored throughout the simulations to ensure energy conservation and trajectory stability. The system was then vertically excited from the ground-state Franck–Condon (FC) region to the first excited singlet state (S_1_), followed by 2000 fs of nonadiabatic molecular dynamics simulations. To ensure that the ultrafast electronic transition processes were accurately captured, the integration time step was set to 0.1 fs for nuclear motion and 0.001 fs for electronic propagation.

To ensure statistical significance, we computed 257 independent trajectories, evaluating the E/Z isomerization based on the final structural configurations. Photoisomerization was defined by a successful rotation around the central C=C double bond leading to a new stable conformer. Of the 257 independent trajectories, 34 completed the full 2000 fs propagation, whereas the remaining trajectories were terminated because the OM2/MRCI iterative electronic-structure solver failed to converge during the dynamics propagation. Among the 34 fully completed trajectories, 10 exhibited successful isomerization, yielding an E → Z photoisomerization quantum yield of around 29.4%. To evaluate whether these premature terminations introduced a structural sampling bias, we analyzed the final dihedral-angle distribution of all terminated trajectories (Figure S3 in Section S4 of the Supporting Information). Although a moderate accumulation is observed around 60–90°, the terminated trajectories span a broad range of dihedral angles, suggesting that the convergence failures are not associated with a single dominant structural preference. Nevertheless, because only 34 trajectories completed the full propagation, the reported quantum yield should be regarded as an estimate based on the available complete trajectories.

Furthermore, to investigate the excited-state decay behaviors and fluorescence dynamics features, time-resolved fluorescence emission spectra were calculated based on the nonadiabatic dynamics results [25,27]. The oscillator strengths, electronic state populations, and energy information required for the spectral simulations were extracted directly from the dynamical trajectories. A Gaussian broadening function was applied to transform the discrete spectral lines into continuous spectra, with the broadening parameter set to 0.05 eV. By analyzing the evolution of the fluorescence signals across different time scales, the fluorescence quenching and wavelength red-shift phenomena accompanying the excited-state decay process can be further elucidated.

## 4. Conclusions

In this study, the high-precision semiempirical OM2/MRCI method combined with NAMD simulations was employed to systematically investigate the ultrafast excited-state dynamics of a salicylaldehyde Schiff base-functionalized visible-light-driven molecular motor M1. The synergistic deactivation mechanism coupling photoisomerization with ESIPT was unravelled at both the atomic and electronic scales.

The results demonstrate that upon photoexcitation, the molecular motor rapidly populates the S_1_ excited state and undergoes structural relaxation along the excited-state potential energy surface, ultimately returning to the ground state via a nonradiative transition through the S_1_/S_0_ conical intersection. The excited-state lifetime of the system is approximately 1677 fs, indicating that this class of visible-light-driven molecular motors possesses a relatively long excited-state evolution time, which facilitates structural rearrangement and unidirectional rotational behavior. Among the fully propagated trajectories, the calculated E → Z photoisomerization quantum yield is approximately 29.4%, confirming that the system can effectively achieve light-driven conformational transformation.

Trajectory analysis reveals that the continuous variation in the central C6–C7–C21–C13 dihedral angle corresponds to the stepwise rotation of the molecular rotor. Within the 500–900 fs time window, the system exhibits pronounced ESIPT, manifested by a significant shortening of the N–H bond length. Concurrently, the rotor experiences a temporary rotational stagnation, which may be indicative of a possible dynamical competition between proton transfer and double-bond rotation. Correlating this with the evolution of the central double bond length and the pyramidalization dihedral angle, it is evident that the proton transfer is accompanied by an enhanced local structural response and electronic redistribution, which subsequently impacts the bond order of the central double bond and its conformational evolution.

Molecular orbital analysis indicates that the HOMO is predominantly distributed over the central conjugated framework and the O-H···N hydrogen-bonding region, whereas the LUMO is largely localized on the stator moiety, displaying a prominent ICT character. The photoinduced redistribution of electron density attenuates the π-bonding nature of the central double bond, thereby lowering the rotational barrier. Simultaneously, it alters the local electronic environment of the hydrogen-bonding region, providing favorable conditions for ESIPT. This suggests that proton transfer may compete with rotor motion during the excited-state relaxation and thus potentially influence the photochemical reaction pathways of the system.

Furthermore, the simulated time-resolved fluorescence emission spectra reveal that the fluorescence signal is rapidly quenched within approximately 37 fs. The substantial discrepancy between the excited-state population lifetime and the fluorescence lifetime suggests that, upon departing from the Franck–Condon (FC) region, the molecule rapidly relaxes to excited-state configurations with markedly reduced transition dipole moments and negligible oscillator strengths. These weakly emissive configurations can be regarded as a “dark” region of the S_1_ potential energy surface. Meanwhile, the emission peak exhibits a noticeable time-dependent red shift, reflecting the rapid relaxation of the excited-state wavepacket toward lower-energy regions of the S_1_ potential energy surface. These results further indicate that the subsequent excited-state dynamics are dominated by nonradiative relaxation processes.

In summary, this work systematically unravels the intrinsic correlations among electron transfer, excited-state proton transfer, double-bond rotation, and nonadiabatic transitions via conical intersections in the salicylaldehyde Schiff base-functionalized molecular motor M1, elucidating the ultrafast dynamical mechanism of its visible-light-driven photoisomerization. This study not only deepens the understanding of the photochemical behaviors of novel visible-light-responsive molecular motors but also provides a robust theoretical foundation for the rational molecular design and performance tuning of highly efficient, long-wavelength-responsive molecular machinery in the future. It should be noted that the present quantum-yield estimation is limited by the relatively small number of fully completed trajectories caused by electronic-structure convergence failures. Although no apparent geometrical bias was observed among the terminated trajectories, increasing the number of fully converged trajectories will further improve the statistical robustness of the calculated quantum yield. Furthermore, in future work, we plan to adopt more robust electronic structure methods, such as XMS-CASPT2 or ADC(2), which are expected to alleviate the convergence difficulties encountered in the present OM2/MRCI calculations and enable larger-scale ensemble dynamics simulations with improved reliability. In addition, given that the present simulations were performed without decoherence corrections, future work will also incorporate decoherence schemes (e.g., the augmented FSSH method or energy-based decoherence approaches) to refine the excited-state lifetime predictions.

## Figures and Tables

**Table 1:** Successful isomerization trajectories and isomerization yields with and without ESIPT.

	Successful Isomerization Trajectories	Isomerization Yield
ESIPT	7	26.9%
No ESIPT	3	37.5%

## Data Availability

The original contributions presented in this study are included in the article/Supplementary Material. Further inquiries can be directed to the corresponding authors.

## Supplementary Materials

The following supporting information can be downloaded at https://www.mdpi.com/article/10.3390/ijms27156627/s1.
