# Supplementary material for: Competing Photoisomerization and Excited-State Intramolecular Proton Transfer in a Visible-Light-Driven Salicylidene Schiff Base Functionalized Molecular Motor: Insights from Ultrafast Nonadiabatic Dynamics Simulations
Source: Int J Mol Sci. 2026 Jul 25;27(15):6627. doi: 10.3390/ijms27156627 (PMC13465398; doi:10.3390/ijms27156627)
Supplement: Supplementary file 1 [file ijms-27-06627-s001.zip › ijms-4442034-supplementary.pdf]

Supporting Information for "Competing Photoisomerization and Excited-State Intramolecular Proton Transfer in a Visible-Light-Driven Salicylidene Schiff Base Functionalized Molecular Motor: Insights from Ultrafast Nonadiabatic Dynamics Simulations"

Xiaojuan Pang [1,2,*], Yue Yan [1], Xueyan Cui [3] and Ningbo Zhang [2,*]

[1] School of Materials Science and Physics, China University of Mining and Technology, Xuzhou 221116, China; ts25180014a31@cumt.edu.cn
[2] School of Mines, China University of Mining and Technology, Xuzhou 221116, China
[3] Department of Science, Henan Institute of Technology, Xinxiang 453003, China; cxy@hait.edu.cn
* Correspondence: xiaojuan.pang@cumt.edu.cn (X.P.); 5864@cumt.edu.cn (N.Z.)

Section S1: Cartesian Coordinates of Representative Geometries...2

Section S2: Conservation of Linear and Angular Momenta and Temperature Evolution.......9

Section S3: Correlation Analysis between ESIPT Timing and E→Z Isomerization Success..11

Section S4: Analysis of Prematurely Terminated Trajectories Due to Electronic-Structure Non-Convergence..14

Section S1: Cartesian Coordinates of Representative Geometries

S1.1 Cartesian coordinates of E configuration

C	-2.7799258266	2.4435729732	-0.7255922777
C	-3.8642410188	1.6024523184	-0.8605432811
C	-3.8081270394	0.3317383957	-0.2530483436
C	-2.6848421717	-0.0242688250	0.5246741611
C	-1.5583949273	0.8377147877	0.6680326118
C	-1.6042524851	2.0386724901	-0.0328570424
C	-0.6547541577	3.1590244917	-0.0567000485
C	-0.4678889353	0.4499815871	1.6042612974
O	-2.5939614672	-1.1866519151	1.1930069250
C	-1.4741213179	4.4078740293	-0.3481368602
C	-2.6625414793	3.8700480881	-1.1748565247
C	-1.9555272039	5.0420187758	0.9466966970
C	1.6332744885	2.0258690426	0.0081340722
C	1.5784017081	0.8297637051	-0.7003527439
C	2.6977559231	-0.0421173787	-0.5613320989
C	3.8234754277	0.3001590941	0.2190510759
C	3.8890981710	1.5664128780	0.8348235081
C	2.8114757748	2.4168332481	0.7045803413
C	2.7048135359	3.8410599343	1.1635025477
C	1.5214787524	4.3939041266	0.3393927095
C	0.6926143919	3.1534958647	0.0388602143
C	2.0091323125	5.0334682657	-0.9504014314
O	2.5977486981	-1.1998198521	-1.2364178152
C	0.4856729726	0.4564086359	-1.6398576130
C	4.9476958859	-0.6272144052	0.3613239139
C	-4.9397161493	-0.5857479907	-0.4007122284
N	-4.9140485718	-1.7295678291	0.1867850675
N	4.9124191153	-1.7676002649	-0.2323143294
C	6.0033241608	-2.6538788678	-0.0991104102
C	-6.0124950455	-2.6056824882	0.0484634716
C	5.7263244346	-4.0193279951	0.0794232162
C	6.7805711142	-4.9183750814	0.1828372281
C	8.0985541320	-4.4726541087	0.0975692662
C	8.3700901429	-3.1202084610	-0.0972214600
C	7.3299988010	-2.2028227352	-0.1965789180
C	-7.3352208963	-2.1436774724	0.1484391461
C	-8.3832161183	-3.0514025961	0.0437193098
C	-8.1234040777	-4.4049984836	-0.1588813180
C	-6.8093408937	-4.8616869210	-0.2464977010
C	-5.7473329225	-3.9724371194	-0.1378372514
H	3.4219457022	-1.7679455963	-1.0845636966
H	-3.4228720175	-1.7470861495	1.0384232665

H	-4.7595041138	1.9096011365	-1.4127551455
H	0.2549091782	-0.2060914002	1.0967804769
H	-0.8988486066	-0.0960389567	2.4555513697
H	0.0563049665	1.3474196509	1.9665804627
H	-0.8783716548	5.1564591566	-0.9436885505
H	-2.4380509314	3.9171138333	-2.2657102683
H	-3.5993103727	4.4363783245	-0.9781107427
H	-2.5953116968	5.9131131880	0.7295348024
H	-1.0932353355	5.3776276551	1.5460228915
H	-2.5310975081	4.3134240996	1.5341524209
H	4.7862531953	1.8629088540	1.3897816262
H	3.6461603505	4.4014129755	0.9715951610
H	2.4795794660	3.8823539564	2.2544493860
H	0.9310752523	5.1429688563	0.9396837832
H	2.5783186902	4.3042288415	-1.5432389146
H	2.6567789165	5.8969715197	-0.7263321576
H	1.1502022512	5.3815423544	-1.5474236062
H	-0.0311305401	1.3601369417	-1.9971027356
H	-0.2427038043	-0.1970855283	-1.1370561808
H	0.9130777145	-0.0877505986	-2.4941332651
H	5.7937043461	-0.2774213276	1.0053642197
H	-5.7825532803	-0.2255717947	-1.0431879549
H	4.6878290319	-4.3525200871	0.1435684928
H	6.5741484312	-5.9846087567	0.3296432613
H	8.9235692233	-5.1888679673	0.1764326521
H	9.4083360680	-2.7773711977	-0.1757878999
H	7.5380894946	-1.1423381416	-0.3713805449
H	-7.5341053916	-1.0824502543	0.3293322194
H	-9.4184643132	-2.7000129965	0.1241626032
H	-8.9546207462	-5.1134957008	-0.2420782046
H	-6.6121769211	-5.9288173675	-0.3994002505
H	-4.7117668530	-4.3142833876	-0.2037751125

S1.2 Cartesian coordinates of Z configuration

C	-1.8709430427	1.3737671462	-1.3495681511
C	-3.2408141452	1.5293189935	-1.2467203876
C	-3.8942583337	1.0012645333	-0.1160049753
C	-3.1519056170	0.2642844068	0.8371356275
C	-1.7400292302	0.1032656304	0.7364950061
C	-1.1068056154	0.7316254178	-0.3391636980
C	0.3011482504	0.6785559251	-0.7707177531
C	-1.0520129453	-0.7746953084	1.7205059636
O	-3.7096811858	-0.3353435751	1.9035489049
C	0.3131351931	0.9654442533	-2.2635644551

C	-0.9931905854	1.7625197831	-2.4986317715
C	1.5060070339	1.8068611965	-2.6837009905
C	2.7513713125	0.2513463629	-0.3332438114
C	3.2024990919	-0.6794439240	-1.2790869856
C	4.5919487786	-0.8682570173	-1.3677979080
C	5.5207879909	-0.2014773635	-0.5298514421
C	5.0326545234	0.6423856619	0.4787784824
C	3.6701407757	0.8375979013	0.5773225468
C	2.9335883036	1.6072406993	1.6305981410
C	1.4622047439	1.1546909061	1.4767679891
C	1.3855519143	0.6233888747	0.0511446956
C	0.5335666620	2.3360911820	1.6971798828
O	5.1590937773	-1.7061823143	-2.2780924296
C	2.2711208457	-1.5130773522	-2.0946615548
C	6.9593989006	-0.4342510059	-0.7062077284
C	-5.3443633913	1.1772168333	0.0214321205
N	-5.9522999814	0.6549420072	1.0226111113
N	7.7795804025	0.1487638900	0.0916139979
C	9.1682014298	-0.0361793197	-0.0289473521
C	-7.3563509938	0.8263551870	1.1061739190
C	9.9613567679	0.6588310589	0.9037046530
C	11.3416332126	0.5375160458	0.8628954348
C	11.9509121438	-0.2715890906	-0.0989276062
C	11.1722950184	-0.9616724434	-1.0238225394
C	9.7896268850	-0.8512972380	-0.9978015366
C	-7.8919204987	1.4907307721	2.2264221846
C	-9.2682566286	1.6201984354	2.3367907429
C	-10.1120529160	1.0964577623	1.3553046535
C	-9.5745781753	0.4270225682	0.2511707460
C	-8.2004217640	0.2915988919	0.1177680903
H	4.4723744080	-2.3277605306	-2.6450721256
H	-4.7065470593	-0.1815049408	1.9136757484
H	-3.8159242064	2.0372680477	-2.0286729281
H	-1.7458128480	-1.5551181732	2.0591849630
H	-0.7352579156	-0.1870065058	2.5992508188
H	-0.1691794340	-1.2422971382	1.2553691091
H	0.2808310915	0.0082143363	-2.8648380279
H	-1.4631125289	1.5051701160	-3.4723435470
H	-0.7981452859	2.8579864688	-2.4721281683
H	2.4449136406	1.2553310731	-2.5581424471
H	1.5564821687	2.7221287321	-2.0748517793
H	1.4020734543	2.0897069670	-3.7442511325
H	5.7355804615	1.1153974655	1.1668427564
H	3.0354074838	2.6998422058	1.4413913086

H	3.3291631530	1.3811944475	2.6436383670
H	1.2372856097	0.3398014242	2.2224656695
H	0.7203977197	2.7687937579	2.6937507009
H	-0.5161912515	2.0271920090	1.6401951660
H	0.7145928057	3.1088620218	0.9341659217
H	2.1258375592	-1.0661312827	-3.0898349705
H	1.3036003271	-1.5870578181	-1.5823947101
H	2.6721268763	-2.5378651259	-2.2188348854
H	7.2273216805	-1.1173375463	-1.5425799878
H	-5.8456114874	1.7717229318	-0.7819904955
H	9.4702786442	1.2879524977	1.6506413452
H	11.9611142964	1.0788644763	1.5882588602
H	13.0427627778	-0.3625928895	-0.1251470414
H	11.6547173419	-1.5956715121	-1.7765504722
H	9.1873379508	-1.3999764911	-1.7316408924
H	-7.2180430247	1.8963860812	2.9845025545
H	-9.6983473279	2.1353461932	3.2041867872
H	-11.1972830283	1.2029850522	1.4526362256
H	-10.2431618135	0.0084341069	-0.5094168672
H	-7.7683950176	-0.2398946031	-0.7364116847

S1.3 Cartesian coordinates of the single proton transfer (SPT) structure

C	-2.7065512071	-0.6867900716	-1.1050504969
C	-4.0860668729	-0.7637521024	-1.1118953493
C	-4.7189403965	-1.6122916740	-0.1812394722
C	-3.9296989120	-2.4091104513	0.6840627178
C	-2.5063052839	-2.3384290717	0.6882624064
C	-1.9158741455	-1.4043918433	-0.1654131317
C	-0.4956174482	-1.1322634667	-0.4361308275
C	-1.7514830853	-3.3104567023	1.5238503425
O	-4.4559802070	-3.2913775035	1.5503224460
C	-0.4209756107	-0.5211593459	-1.8265336231
C	-1.8319644366	0.0738419631	-2.0518218171
C	0.6204333264	0.5816690618	-1.9150252906
C	1.9506806319	-1.2638966489	0.1561632145
C	2.6000268542	-1.8722744885	-0.8935126289
C	4.0648878425	-1.8561643938	-0.9114453998
C	4.7790038104	-1.2637583377	0.2137140918
C	4.0511364063	-0.7569516000	1.3410866325
C	2.6926835737	-0.7730188162	1.3008620779
C	1.7438447770	-0.3911242207	2.3933270543
C	0.3940693215	-1.0157675020	1.9731891919
C	0.5198696187	-1.2010120922	0.4686133183
C	-0.7426461557	-0.0776233424	2.3426805499

O	4.7073217859	-2.3477727198	-1.8713322253
C	1.9373366317	-2.6294577867	-1.9860717781
C	6.1773544682	-1.2751351139	0.2274645466
C	-6.1782034149	-1.6818638896	-0.1495750858
N	-6.7594518834	-2.4606066254	0.6943139150
N	6.8784633378	-1.7815759077	-0.7975793499
C	8.2796116750	-1.9056605633	-0.8406256988
C	-8.1694802700	-2.5033177136	0.7367922244
C	8.8273777997	-2.7433167887	-1.8298330711
C	10.2057070850	-2.9056080287	-1.8960929481
C	11.0377080722	-2.2422161005	-0.9978735347
C	10.4923682952	-1.4037978502	-0.0306724615
C	9.1152360284	-1.2213856944	0.0549395518
C	-8.9584190737	-2.4052946711	-0.4199182546
C	-10.3430677400	-2.4719784209	-0.3114765046
C	-10.9434349990	-2.6295096255	0.9360526156
C	-10.1587323598	-2.7417886084	2.0809766812
C	-8.7731341272	-2.6915017617	1.9925108031
H	6.3127607475	-2.1867263774	-1.5817914564
H	-5.4657458670	-3.2679687096	1.5025537089
H	-4.6842783651	-0.1864996191	-1.8267583528
H	-1.6078989034	-2.9070898288	2.5416310873
H	-0.7700490890	-3.5154096192	1.0683935348
H	-2.3232386780	-4.2451083421	1.6015330775
H	-0.2162881082	-1.3101088332	-2.6075067106
H	-2.1702771945	-0.0511606355	-3.1038429389
H	-1.8494574724	1.1580125081	-1.7969935220
H	0.5487931196	1.0778894463	-2.8973165984
H	1.6320202754	0.1767585557	-1.7990793642
H	0.4491288149	1.3292616191	-1.1249226180
H	4.5986064100	-0.3851779499	2.2169009649
H	1.6658788451	0.7199447820	2.4552926825
H	2.0840079330	-0.7770832074	3.3803949601
H	0.2604771100	-2.0090070907	2.4930361122
H	-0.6412069513	0.8740625477	1.7978726414
H	-0.7136764740	0.1274034201	3.4260770195
H	-1.7141039222	-0.5204971557	2.0952287603
H	2.6098646192	-3.4245332581	-2.3363491437
H	1.7221906487	-1.9620025496	-2.8405123717
H	0.9951793315	-3.0722887786	-1.6253320234
H	6.7121717842	-0.8542303539	1.1073337459
H	-6.7156977160	-1.0295480467	-0.8832247192
H	8.1677909244	-3.2596870771	-2.5314717743
H	10.6375002640	-3.5607831323	-2.6605363056

H	12.1223146848	-2.3783942524	-1.0541986424
H	11.1500921261	-0.8756596454	0.6690966327
H	8.6975329439	-0.5522800716	0.8130846230
H	-8.4863936776	-2.3049385351	-1.4037588110
H	-10.9641140694	-2.4067529718	-1.2121617395
H	-12.0351470693	-2.6721002269	1.0143656294
H	-10.6366567905	-2.8734654578	3.0589272275
H	-8.1446171577	-2.7736451449	2.8826211839

S1.4 Cartesian coordinates of the double proton transfer (DPT) structure

C	-2.4855717703	1.9878561270	-1.2962824984
C	-3.6035198915	1.2157906810	-1.4256268571
C	-3.9287056172	0.3166928849	-0.3694278986
C	-3.0600390612	0.1533940809	0.7948027109
C	-1.8293997337	0.9235072752	0.8431477409
C	-1.5548355827	1.7522986533	-0.2135042708
C	-0.5518684343	2.8130345191	-0.1999478589
C	-0.9801772286	0.8810500146	2.0708138981
O	-3.3952457075	-0.6289505142	1.7255071286
C	-1.2025744632	3.9751679488	-0.9298461144
C	-2.0668057948	3.2431610299	-2.0049608978
C	-2.0935773687	4.8090268659	-0.0231013892
C	1.5669958709	1.5727784190	0.3459628811
C	1.5812878777	0.4771372583	-0.4975506656
C	2.7973775723	-0.3697815425	-0.5340682704
C	3.9104972766	-0.0737935116	0.3633672514
C	3.8332107812	1.0935266169	1.2003411229
C	2.7154333119	1.8749290189	1.1753374960
C	2.5370585021	3.2010287130	1.8583305630
C	1.3358705263	3.8611520850	1.1519638896
C	0.6897909479	2.7374407578	0.3551698057
C	1.7825453982	5.0206181066	0.2740385518
O	2.8837534101	-1.3254216196	-1.3299530812
C	0.5157583752	0.1199626592	-1.4674680009
C	5.0534899059	-0.8722845200	0.4056877530
C	-5.1162005122	-0.4339066793	-0.4171692771
N	-5.4897646361	-1.2082811139	0.6013384471
N	5.2570252500	-1.9565029402	-0.3786818908
C	6.5577050156	-2.5136554758	-0.4464768015
C	-6.5584769750	-2.1247490308	0.4135108914
C	7.2205109776	-2.8556564212	0.7485198912
C	8.5286153244	-3.3129917027	0.6804268411
C	9.1807194672	-3.4340634194	-0.5481624797
C	8.5078043266	-3.1329624436	-1.7364633295

C	7.2057266493	-2.6611763576	-1.6897558181
C	-7.8045494902	-1.9165326849	1.0143650949
C	-8.8099353713	-2.8582917662	0.8050968174
C	-8.5696967050	-3.9733534665	0.0044097384
C	-7.3330024485	-4.1608576767	-0.6077997113
C	-6.3127878708	-3.2387500212	-0.4045066715
H	4.5551015450	-2.1748259570	-1.0973931085
H	-4.8133121478	-1.3163354362	1.4010348239
H	-4.3026037145	1.3392100283	-2.2632712184
H	-0.7518750818	1.9187614682	2.3906488905
H	-0.0325401096	0.3655489164	1.8705872969
H	-1.5107939311	0.3590840673	2.8751065674
H	-0.4224976293	4.6295862283	-1.4102958961
H	-1.4532125818	3.0136130329	-2.9060494635
H	-2.9456529785	3.8510036579	-2.3134786387
H	-2.5073741384	5.6611379609	-0.5901355116
H	-1.5136917479	5.1961640527	0.8262567743
H	-2.9244440064	4.1986686262	0.3541908387
H	4.7123937747	1.3751347078	1.8039826822
H	3.4591712442	3.8199145922	1.7754021628
H	2.3180093351	3.0369617913	2.9382883127
H	0.5969458225	4.2444012256	1.9246554015
H	2.5005034020	4.6801642072	-0.4859962018
H	2.2571520278	5.8024602646	0.8903051054
H	0.9161017960	5.4655317974	-0.2407751835
H	0.9129884815	-0.6445902573	-2.1499764892
H	0.2258531278	1.0151392294	-2.0446955212
H	-0.3742660645	-0.2791694497	-0.9602230293
H	5.8642651709	-0.6030944341	1.1253517627
H	-5.7793825754	-0.3587576457	-1.3076315200
H	6.7033117763	-2.7773852597	1.7067106055
H	9.0592288417	-3.5804564957	1.6028002889
H	10.2132014001	-3.7918933536	-0.5826969682
H	9.0143897751	-3.2568021786	-2.6942916884
H	6.6720154731	-2.4142632421	-2.6116945820
H	-7.9681098730	-1.0418335727	1.6481199556
H	-9.7939115621	-2.7243956390	1.2680935154
H	-9.3613092519	-4.7187377786	-0.1428141461
H	-7.1626174853	-5.0378827704	-1.2415307511
H	-5.3353122938	-3.3723431524	-0.8755938607

Section S2: Conservation of Linear and Angular Momenta and Temperature Evolution

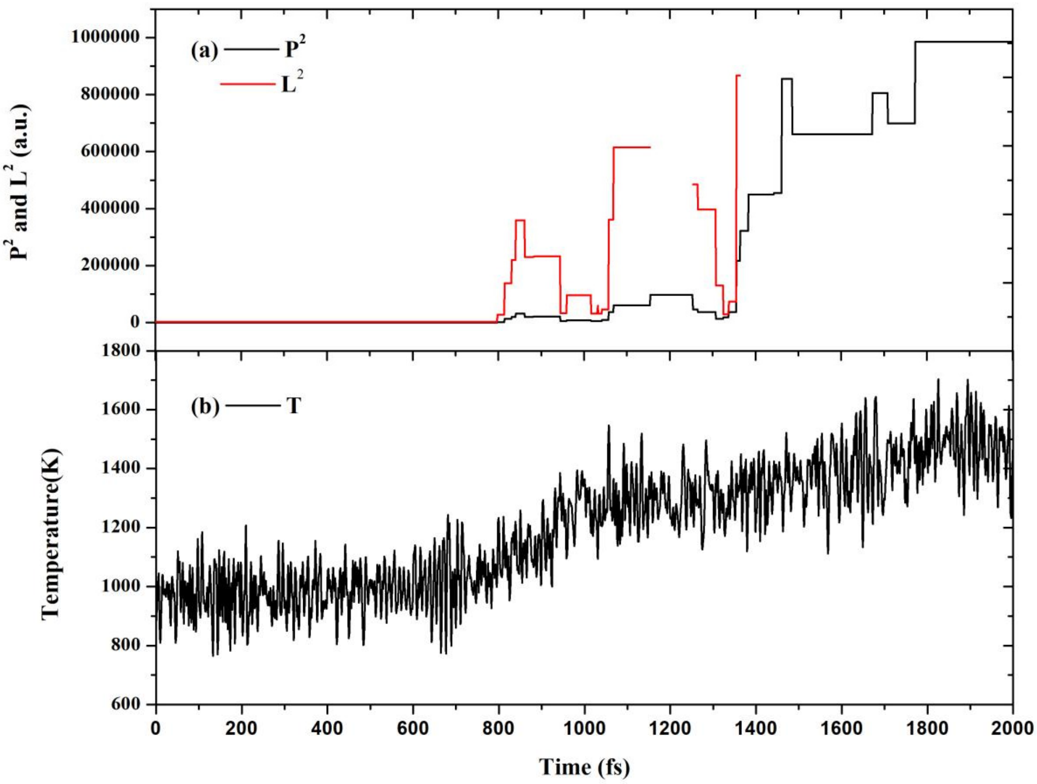

Figure S1. Time profiles of the geometric parameters (a) P^2, L^2 and (b) T during the 2000 fs nonadiabatic dynamics simulation (definitions in text).

To validate the stability of the NVE dynamics and to investigate the effect of velocity rescaling during surface hopping, we analyzed the time evolution of the squared total linear momentum (P^2), the squared total angular momentum (L^2), and the instantaneous temperature along a representative trajectory that undergoes a surface-hopping event at 798 fs. The results are presented in Figure S1.

As shown in Figure S1(a), prior to the surface-hopping event at 798 fs, P^2 remains strictly zero, confirming that the overall translation has been successfully removed (NOTRA = T in the dynvar.in input file). However, at the exact moment of the hop (798 fs), P^2 exhibits a sudden but small jump from ~0 to ~0.1 a.u., indicating that the velocity rescaling along the nonadiabatic coupling vector (NACV) introduces a small net linear momentum. This suggests that the NACV direction contains a component that does not perfectly pass through the molecular center of mass. After this transient jump, P^2 returns to zero and remains conserved for the remainder of the trajectory.

Figure S1(a) shows the evolution of L^2 with red solid line. Prior to the hop, L^2 is conserved near zero. At 798 fs, however, L^2 undergoes a dramatic jump from ~0 to approximately 100,000 a.u., and then remains conserved at this new level for the rest of the trajectory. This observation

provides direct numerical evidence that the velocity rescaling along the NACV—required to conserve total energy during the downward hop—introduces a substantial "sudden boost" to rotational motion. The magnitude of the L^2 jump reflects that the rescaling predominantly excites rotational degrees of freedom, consistent with the reviewer's suggestion that such large energy-gap hops may introduce dynamical instabilities.

Figure S1(b) displays the instantaneous temperature evolution along the same trajectory. The temperature fluctuates around a mean value of approximately 1198 K with a standard deviation of about 208 K throughout the 2000 fs simulation. Prior to the hopping event, the temperature exhibits relatively stable oscillations around 1000 – 1200 K. At 798 fs, coincident with the jumps in P^2 and L^2, a sharp temperature increase to approximately 1800 K is observed. Following this peak, the temperature gradually relaxes and stabilizes around 1300 – 1500 K for the remainder of the trajectory. This elevated temperature is a direct consequence of the NVE ensemble employed: the photoexcitation energy (~3.0 eV, corresponding to 415 nm absorption) is fully retained within the system and redistributed into vibrational modes during the excited-state dynamics. The temperature jump at the hopping event further reflects the sudden kinetic energy injection resulting from the velocity rescaling along the NACV.

Importantly, no thermostat was applied during the dynamics—the system evolves under the microcanonical (NVE) ensemble with total energy conservation, as indicated by VS = F (velocity scaling switched off) in the dynvar.in input file. The observed jumps in P^2 and L^2 at the surface-hopping event reveal a significant limitation of the standard velocity rescaling scheme: while necessary for energy conservation within the FSSH framework, it inevitably introduces artificial momentum when the NACV does not align perfectly with the molecular center of mass. This issue is particularly pronounced when the energy gap is large (40 kcal/mol in this case), as the required kinetic energy adjustment becomes substantial. The resulting artificial injection of translational and rotational energy likely contributes to the trajectory instabilities and convergence failures observed in a subset of our simulations, and may affect the quantitative accuracy of the excited-state lifetimes and quantum yields derived from these trajectories. Therefore, the reported quantum yield (29.4%) and excited-state lifetime (~1677 fs) should be regarded as estimates that would benefit from validation with more robust electronic structure methods and improved surface-hopping implementations.

Section S3: Correlation Analysis between ESIPT Timing and E→Z Isomerization Success

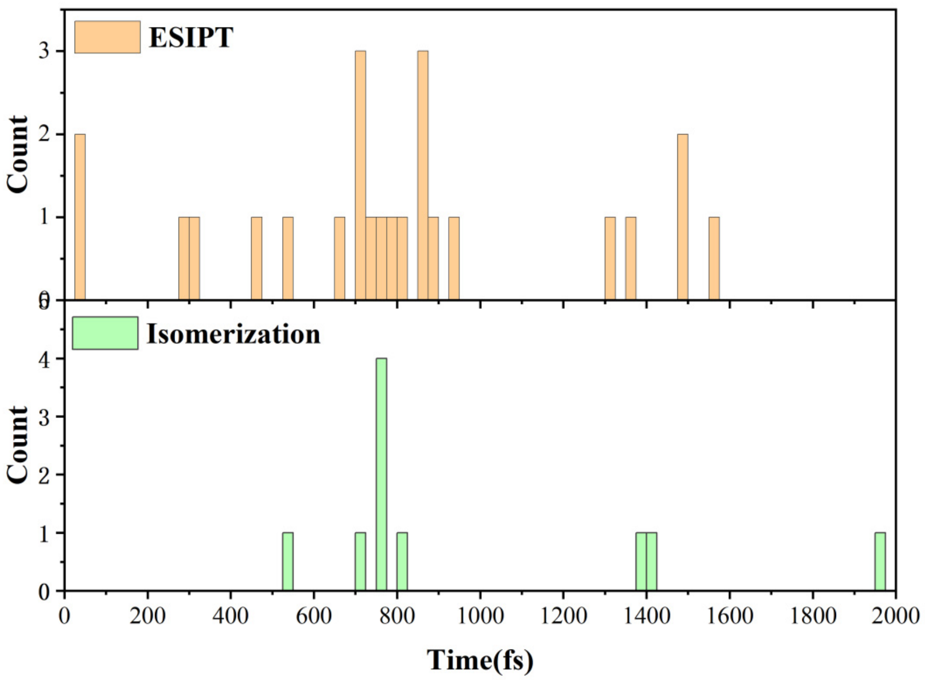

Figure S2. Statistical distribution of ESIPT and E→Z isomerization events as a function of time. The histogram shows the number of trajectories that underwent ESIPT (yellow bars) and the number of trajectories that successfully completed isomerization (green bars) at each time bin, compiled from all 25 fully propagated trajectories. The time resolution is 100 fs.

This statistical overview provides a complementary perspective to the trajectory-specific correlation analysis presented in Table S1. As shown in Figure S2, ESIPT events are broadly distributed across the simulation window from 300 fs to 1600 fs, with a notable concentration around 700–800 fs where multiple trajectories undergo proton transfer simultaneously. In contrast, successful isomerization events are more sparsely distributed and occur primarily within the 500–1500 fs range, with a peak around 700–800 fs that coincides with the most active ESIPT period. This temporal overlap between the two processes at 700–800 fs suggests that ESIPT and isomerization can occur concurrently, yet the fact that successful isomerization events are fewer than ESIPT events at most time points implies that proton transfer does not guarantee rotational success. Notably, no isomerization events are observed beyond 1500 fs, whereas ESIPT continues to occur up to 1550 fs, indicating that late-stage proton transfer is unlikely to contribute to productive photoisomerization. The early ESIPT events occurring at 30–100 fs (Trajectories 10 and 24) are followed by no subsequent isomerization in those trajectories, further supporting the notion that early proton transfer may trap the molecule in a local minimum and divert it away from the rotational pathway. Overall, the statistical distribution is consistent with the conclusion drawn from the trajectory-specific analysis: ESIPT and E→Z isomerization are dynamically

competing processes, where proton transfer tends to hinder rather than promote successful photoisomerization.

Table S1. Summary of proton transfer (ESIPT) events and E→Z isomerization outcomes for each of the 25 fully propagated nonadiabatic dynamics trajectories. The table lists the time (in femtoseconds) at which excited-state intramolecular proton transfer occurs (if any), and the time at which E→Z isomerization is completed (defined as the moment when the C6–C7–C21–C13 dihedral angle surpasses 90°). Empty entries indicate that the corresponding event did not occur within the 2000 fs simulation window. Trajectories highlighted in blue bold indicate those that underwent both ESIPT and E→Z isomerization. This compilation enables a direct assessment of whether ESIPT precedes, coincides with, or follows the rotational motion, and whether its occurrence correlates with successful photoisomerization.

Trajectory number	ESIPT(fs)	Isomerization(fs)
1	810	
2	**1490**	**700**
3	**540**	**770**
4	315	
5	700	
6	870	
7	450	
8	**1310**	**810**
9	**850**	**770**
10	30	
11	**1350**	**750**
12	700	
13	1550	
14	650	
15	870	
16	770	
17	**1491**	**1390**
18	730	
19	**310**	**1950**
20	890	
21	930	
22	290	
23	780	
24	30	
25	710	

26	410	
27		750
28		1420
29		540

Table S1 summarizes the timing of ESIPT events and E→Z isomerization completion for all 25 fully propagated trajectories, where isomerization completion is defined as the moment when the C6–C7–C21–C13 dihedral angle first exceeds 90°. Trajectories that accomplished both processes are highlighted in bold. As shown in the table, ESIPT events are distributed across a broad temporal window ranging from 30 fs to 1550 fs, while successful isomerization events occur between 540 fs and 1950 fs, with no simple temporal correlation between the two. Notably, Trajectory 2 completes isomerization at 700 fs but does not undergo ESIPT until 1490 fs, demonstrating that proton transfer is not a prerequisite for double-bond rotation. Statistically, among the 23 trajectories that underwent ESIPT, 8 successfully isomerized (success rate ~34.8%), whereas among the 2 trajectories without ESIPT, 1 successfully isomerized (success rate 50.0%). Although the small number of non-ESIPT trajectories precludes rigorous statistical testing, this trend is consistent with the trajectory-level observation that the dihedral angle exhibits transient stalling during the 500–900 fs window where ESIPT events are most concentrated (Figure 4), suggesting that proton transfer temporarily impedes the rotational progression. The temporal order of the two events also varies across trajectories—some exhibit isomerization prior to ESIPT (Trajectory 2) while others show the opposite sequence (Trajectory 19)—further supporting that ESIPT and double-bond rotation are dynamically competitive rather than sequentially coupled. Importantly, 8 trajectories accomplish both processes, indicating that ESIPT does not completely suppress isomerization but modestly reduces its efficiency. Overall, the correlation analysis reveals a slightly negative correlation between ESIPT and rotational success, leading us to conclude that proton transfer tends to hinder rather than promote E→Z photoisomerization, consistent with the stalling behavior observed in the trajectory analysis (Figure 4).

Section S4: Analysis of Prematurely Terminated Trajectories Due to Electronic-Structure Non-Convergence

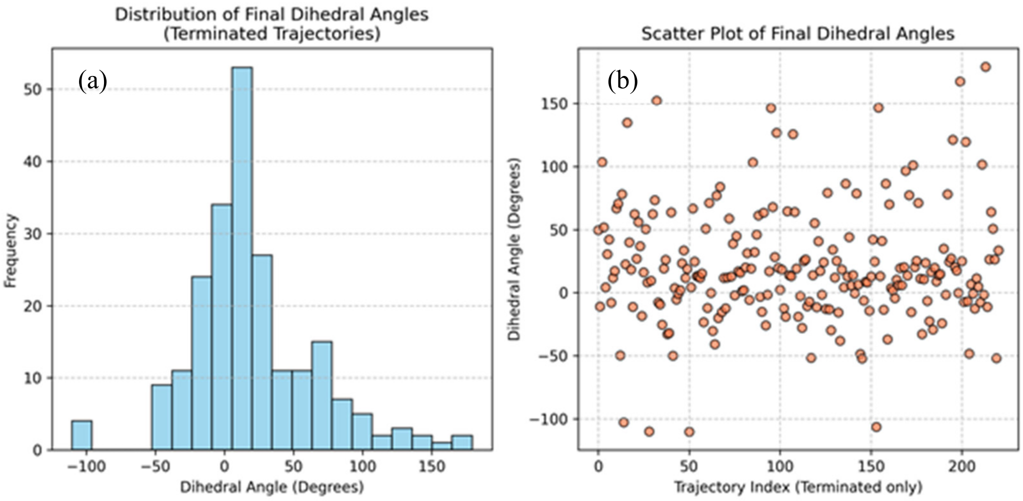

Figure S3. Distribution of final C6–C7–C21–C13 dihedral angles for the trajectories that terminated prematurely due to electronic-structure non-convergence. (a) Histogram showing the frequency distribution of final dihedral angles. (b) Scatter plot of final dihedral angles versus trajectory index.

To evaluate whether the premature termination of trajectories introduced a structural sampling bias, we analyzed the final C6–C7–C21–C13 dihedral angles for all trajectories that did not complete the full 2000 fs propagation because of convergence failures in the OM2/MRCI electronic-structure calculations. A total of 223 trajectories terminated prior to the designated simulation time, and their final geometries were collected for statistical analysis. As shown in Figure S3(a), the final dihedral angles of the terminated trajectories are broadly distributed across a wide range from approximately -100° to 180°, with a notable accumulation around -50°–90° as indicated by the pronounced peak in the histogram. This suggests that a significant fraction of the prematurely terminated trajectories converge to a conformation near the early-to-intermediate stage of rotation, where the central dihedral angle has partially twisted but has not yet reached the conical intersection region. It is difficult to ascertain whether the incomplete trajectories would have preferentially led to successful or failed isomerization, as the majority terminated at dihedral angles corresponding to the early-to-intermediate stage of rotation.Therefore, the reported value should be interpreted as an estimate with associated statistical uncertainty rather than a fully converged benchmark.

The scatter plot in Figure S3(b) further reveals that the final dihedral angles are not clustered at a single narrow range but instead show a relatively broad spread across the trajectory index,

although the density is higher in the -50°–90° region. This distribution implies that the convergence failures may be more frequent in a specific conformational region where the electronic structure calculations encounter numerical difficulties. Several factors could contribute to this observation. First, the -50°–90° region may correspond to a region of the S_1 potential energy surface with strong nonadiabatic couplings or small energy gaps, where the multireference wavefunction becomes particularly sensitive to the molecular geometry, increasing the likelihood of convergence failure in the MRCI iterations. Second, this angular range may coincide with the onset of ESIPT (as observed in Figure 4 of the main text, where proton transfer occurs primarily within the 500–900 fs window, during which the dihedral angle oscillates around 65°), suggesting that the electronic structure changes associated with proton transfer may further complicate the convergence of the MRCI procedure. Nevertheless, the fact that the terminated trajectories span the entire range of dihedral angles from −100° to 180° indicates that the convergence failures are not exclusively confined to a single conformation.
